# Fish and Marine Omega-3 Polyunsatured Fatty Acid Consumption and Incidence of Type 2 Diabetes: A Systematic Review and Meta-Analysis

**DOI:** 10.1155/2013/501015

**Published:** 2013-09-08

**Authors:** Ming Zhang, Eliane Picard-Deland, André Marette

**Affiliations:** ^1^Department of Medicine, Faculty of Medicine, Cardiology Axis of the Institut Universitaire de Cardiologie et de Pneumologie de Québec (Hôpital Laval), Québec, QC, Canada G1V 4G2; ^2^The Institute of Nutraceuticals and Functional Foods, Laval University, Québec, QC, Canada G1V 4G2

## Abstract

*Objective.* To examine the association between fish and marine long-chain omega-3 polyunsaturated fatty acid (LC n-3 PUFA) consumption and incidence of type 2 diabetes (T2D) in prospective cohort studies. *Methods.* Meta-analytic procedures were used to estimate the relative risk (RR) using random effects or fixed effects generic inverse variance model. Publication bias and study heterogeneity were assessed using Egger's test and I^2^ statistic. *Results.* We found no significant association between the intake of fish/seafood (pooled RR: 1.04; *P* = 0.63, 95% CI: 0.9 to 1.2, 549, 955 participants) or marine LC n-3 PUFA (pooled RR: 1.08, *P* = 0.39, 95% CI: 0.90 to 1.30, 346, 710 participants) and T2D risk. Significant study heterogeneity was observed in fish/seafood and marine LC n-3 PUFA studies (*P* < 0.00001). Subgroup analysis revealed no obvious sources for high heterogeneity. We also found a significant protective effect of oily fish intake on T2D risk (pooled RR = 0.89, *P* = 0.005, 95% CI: 0.82 to 0.96). Dose-response analysis suggested that every 80 g per day intake of oily fish may reduce 20% risk of T2D. *Conclusion.* We found no significant effect of fish/seafood or marine LC n-3 PUFA intake on risk of T2D but a significant effect of oily fish intake on risk of T2D.

## 1. Introduction

Type 2 diabetes (T2D) is the most common form of diabetes and its prevalence is steadily increasing by about 6.4% annually worldwide [1]. However the etiology of T2D is still unclear. Both genetic and environmental factors have been shown to be involved in T2D incidence. It is well established that obesity and low physical activity are high risk factors for T2D [2]. Importantly, dietary factors are also risk modulators for this disease [3]. Meat consumption [4] and western diet [5] have been linked to increased T2D risk, while carbohydrates, fiber [6, 7], green leaf vegetables [8], and dietary pattern [9] have all been reported to reduce T2D risk. Interestingly, high intake of fish has been associated with a reduced incidence of mortality due to cardiovascular disease [10], which shares many common risk factors with T2D. Currently, the association between fish intake and T2D risk is still not fully understood. 

It has been reported that in countries with a high prevalence of obesity, the incidence of T2D is significantly reduced with high fish and seafood consumption (2.5 ± 1.8% versus 0.9 ± 0.7%; *P* = 0.007 and 11.0 ± 3.9% versus 6.2 ± 4.1%; *P* = 0.041 for the 20 to 44 and 45 to 64 year age groups, resp.) [11]. The associations between high intake of fish/seafood and marine long-chain omega-3 polyunsaturated fatty acid (LC n-3 PUFA) and incidence of T2D have been investigated in several prospective cohort studies [12–22]. However the conclusions are inconsistent. Three cohort studies showed reduced risk of T2D with high intake of fish [12, 13, 16], while seven studies showed no difference or increased risk of T2D with high fish intake [14, 15, 17, 18, 20–22]. Fish types, cooking methods, selenium, mercury, and other environmental contaminants in fish were potential factors influencing the results [12]. High intake of marine LC n-3 PUFA was reported to reduce the risk of T2D in two cohort studies [13, 19] but to increase T2D risk in four others [14, 15, 18, 20]. To clarify these associations, we conducted a meta-analysis of fish/seafood and marine LC n-3 PUFA intake and T2D incidence in prospective cohort studies.

## 2. Methods

### 2.1. Search Strategy

We searched PubMed, OViD, and EMBASE databases from their respective launch dates to May 2013. The searching subject terms in heads, abstracts, or texts were specified to T2D, fish, seafood, omega-3 fatty acid, follow-up, prospective studies, and cohort studies. Cross-references of studies or reviews that were included in the analysis were also examined.

### 2.2. Study Selection and Assessment

The eligible studies had to meet the following criteria: (1) to be a prospective cohort design and study the association between fish/seafood, omega-3 fatty acid intake, and the incidence of T2D; (2) risk ratios or odds ratios have to be available with 95% confidence intervals (CI), or otherwise the case numbers and participant numbers in both highest and lowest intake groups should be available; (3) the method of dietary assessment had to be reported, and participants should consume either fish/seafood that includes fish (such as salmon, tuna, trout, and tilapia) and shellfish (such as shrimp, crab, and oysters), and/or LC n-3 PUFA (e.g., EPA and DHA); and (4) the participants at baseline were not already diagnosed as being diabetic.

We assessed all studies for quality using a scoring system that accounted for participants (1 point if a power calculation had been conducted to give the numbers of participants needed to detect an effect of fish/seafood intake on risk of T2D and 1 point for appropriate inclusion and exclusion criteria), outcome (1 point if T2D was confirmed by clinical criteria or blood tests), assessment of diet (1 point if a validated FFQ was used), relative risk (RR) adjustment for seven T2D risk factors (age, BMI, family history of diabetes, physical activity, vegetable intake, fruit intake, and meat intake) (1 point for each risk factor), and RR adjustment for other factors such as energy intake (1 point). This scoring system was designed with reference to [8]. Studies were assessed as high quality if they had a score of 9–12 points and moderate quality if they had a quality score of 5–8 points.

### 2.3. Data Extraction

We extracted data on the diagnosis of T2D, intake of fish/seafood and marine LC n-3 PUFA, the adjusted RR, and 95% CI. For those with odds ratio (OR) data, we converted OR to RR using a previously published formula [23], and the corresponding CI Values were also converted. For studies that had separate results for men and women, we generated a pooled RR for the total population. We also extracted other information from each eligible paper, including the country of the study, the sample size (participants' numbers) at baseline, the age of participants, the method of assessing diabetes status, follow-up years, the types of fish/seafood and marine LC n-3 PUFA they measured, and the highest and lowest intake amounts of fish/seafood or marine LC n-3 PUFA (Table 1). M. Z and E. P. D conducted study selection, data extraction, and quality assessment independently, with disagreements resolved by consensus after discussion with A. M.

### 2.4. Statistical Methods

We transformed the RRs by using their natural logarithms and calculating standard errors and corresponding CI. Heterogeneity was assessed with the I^2^ statistic. We calculated the summary RRs and 95% CI for the highest versus the lowest intake according to Dersimonian and Laird for the random effects generic inverse variance model [24] when heterogeneity was found significant (*P* < 0.05); otherwise the fixed effects generic inverse variance model was used according to Hedges and Olgin. We also conducted meta-analysis of stratified samples according to gender (men and women) and fish/seafood types (e.g., shellfish, oily fish, and lean fish). The publication bias was assessed by the asymmetry of funnel plot and Egger's regression test [25]. The meta-analysis was conducted by Review Manager 5.1 (The Nordic Cochrane Centre, The Cochrane Collaboration). A two-tailed *P* < 0.05 was considered as statistically significant, and 95% confidence intervals were quoted where available.

Dose-response analysis for consumption of fish/seafood and marine LC n-3 PUFA was conducted by using a previously reported method [10]. We included intake, adjusted RR, and CI from all related studies except one [17] that had no quartile information. The median or mean level of fish or marine omega-3 fatty acid intake was assigned to the corresponding RR for each study. For those reported ranges of intake, we estimated the mean intake in each category. When the lowest dose was open-ended, we set the lower boundary to zero. When the highest dose was open-ended, we assumed that the interval length was the same as the adjacent interval [4]. For publications that provided servings per day for fish intake, we transformed them into g/day by 100 g per serving [10]. Linear regression was used to estimate the relationship between total fish, oily fish and marine omega-3 fatty acid intake, and incidence of T2D. STATA 11.0 was used for dose-response analysis.

Subgroup analysis was conducted based on ethnicity (Asian versus US/European), length of follow-up (<10 years versus ≥10 years), assessment of T2D (confirmed by physician/phone interview/hospital records versus confirmed by standard criteria/plasma glucose level), sample size (<10,000 versus ≥10,000), and study quality score (high quality (9–12 points) versus moderate quality (5–8 points)), as these factors are possible sources of study heterogeneity. The Mann Whitney *U* test was used to calculate the significance of differences within subgroups and to detect factors contributing to heterogeneity. 

## 3. Results

### 3.1. Selection of Studies

**Figure 1:**
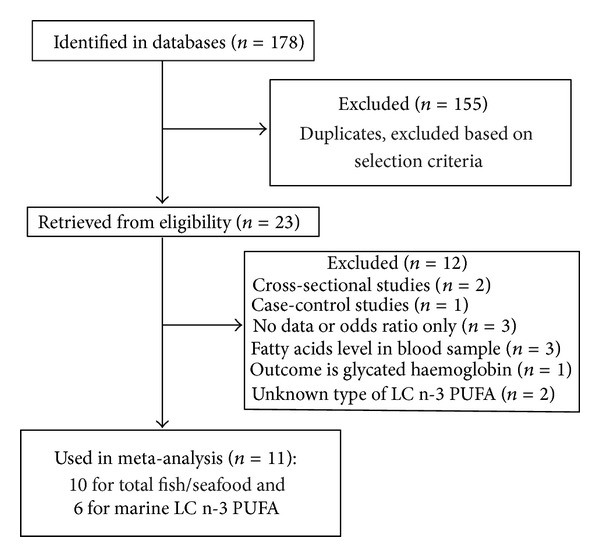
Process of study selection.

We identified 178 candidate publications related to fish/seafood intake and risk of T2D in prospective cohort studies through searching PubMed, OViD, and EMBASE databases. Among them, 155 articles were excluded based on our inclusion criteria and duplicated reports. A further 12 studies were excluded with more specific criteria, including cross-section, studies, case-control studies, those without original data or with odds ratio only, those with fatty acid levels in blood samples, those with outcomes reported as glycated haemoglobin, and those with unknown type of LC n-3 PUFA (Figure 1). The remaining 11 studies were assessed in the current meta-analysis. 10 prospective studies (549,955 participants at baseline) [12–18, 20–22] were used for measuring the relationship between fish/seafood intake and risk of T2D (Table 1). Six prospective studies [13–15, 18–20] (346,710 participants at baseline) were selected for assessing the association between marine LC n-3 PUFA intake and risk of T2D quality (Table 1). 

### 3.2. Fish/Seafood Intake and Risk of T2D

**Figure 2:**
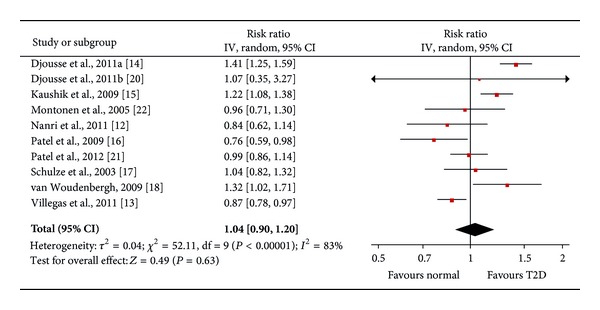
Forest plot of the meta-analysis for fish/seafood intake and incidence of type 2 diabetes.

No significant association between high fish/seafood intake and T2D incidence was observed (pooled RR: 1.04; 95% CI: 0.890 to 1.20; *P* = 0.63) (Figure 2). However, there was a significant study heterogeneity (*I* ^2^ = 83%, *P* < 0.00001). Subgroup analysis showed no clear sources of this high heterogeneity (ethnicity, *P* = 0.18; follow-up years, *P* = 0.17; assessment of T2D, *P* = 0.18; sample size, *P* = 0.51; study quality, *P* = 0.18, MWU test) (Table 2). Two studies using Asian populations showed a beneficial effect of fish/seafood intake on risk of T2D (pooled RR = 0.87, *P* = 0.006), but six studies of western populations demonstrated no significant effect of fish/seafood intake on T2D risk (pooled RR = 1.10, *P* = 0.22). Four studies with less than 10 years of follow-up showed a protective effect of fish/seafood intake against development of T2D (Pooled RR = 0.91, *P* = 0.11), while six studies with more than 10 years of follow-up indicated an increased risk of T2D with high fish/seafood intake (Pooled RR = 1.17, *P* = 0.04). The high quality studies demonstrated a significant protective effect of high fish/seafood intake on incidence of T2D (pooled RR = 0.87, 95% CI: 0.78 to 0.96, *P* = 0.006), while the moderate quality studies showed no obvious effect of fish/seafood intake on risk of T2D (pooled RR = 1.10, 95% CI: 0.95 to 1.04, *P* = 0.22). Egger's regression test and funnel plot showed no significant publication bias (Egger's *P* > 0.05). Dose-response analysis for fish/seafood intake showed no significant linear relationship between fish/seafood intake and risk of T2D (*R* ^2^ = 0.11, *P* for regression = 0.076). 

### 3.3. Fish Types Intake and Risk of T2D

**Figure 3:**
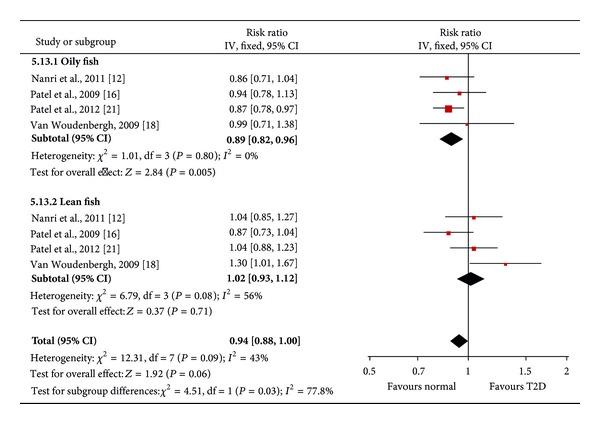
Forest plot of the meta-analysis for oily fish and lean fish intake and incidence of type 2 diabetes.

**Figure 4:**
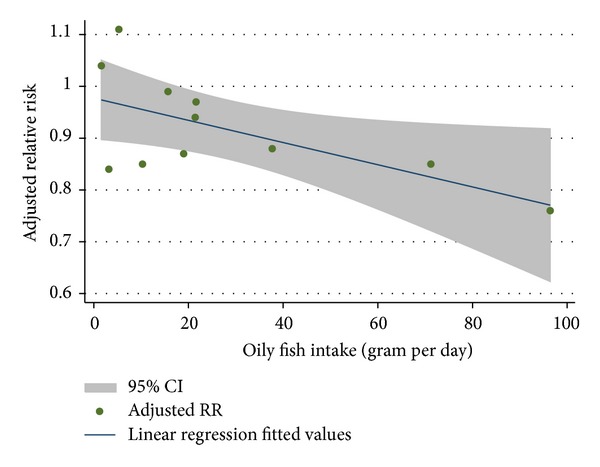
Linear regression of adjusted RR of T2D versus oily fish intake. The upper and lower bands denote the 95% confidence interval on mean of the predicted value, *R* ^2^ = 0.40.

**Figure 5:**
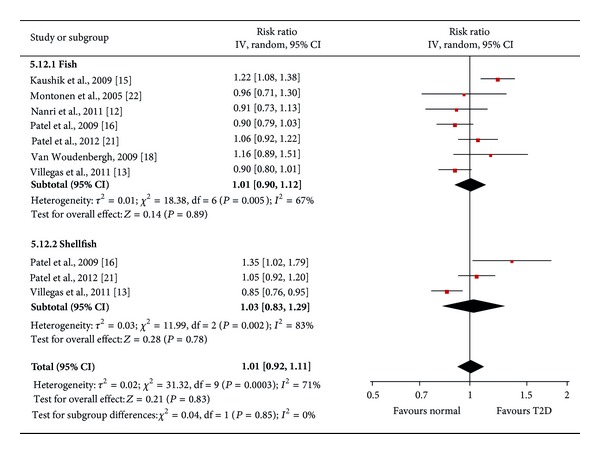
Forest plot of the meta-analysis for fish and shellfish intake and incidence of type 2 diabetes.

We also conducted meta-analysis of stratified samples based on fish/seafood types (oily fish and lean fish, fish, and shellfish). We found a significant protective effect of high oily fish intake on T2D risk (pooled RR = 0.89, *P* = 0.005, 95% CI: 0.82 to 0.96, 103,949 participants), but lean fish intake had no significant effect on T2D risk (pooled RR = 1.02, *P* = 0.66, 95% CI: 0.93 to 1.12) (Figure 3). In order to better understand the effect of oily fish intake on risk of T2D, we also conducted a dose-response analysis by linear regression (Figure 4). We found that 80 g/day oily fish intake may reduce 20% risk of T2D. Meanwhile, we observed no significant effect of high consumption of fish (including oily fish and lean fish, fresh or canned) (pooled RR = 1.01, *P* = 0.89, 95% CI: 0.90 to 1.12) or shellfish (pooled RR = 1.03, *P* = 0.78, 95% CI: 0.83 to 1.29) on incidence of T2D (Figure 5). Egger's regression test and funnel plot showed no significant publication bias (Egger's *P* > 0.05). 

### 3.4. Marine LC n-3 PUFA Intake and Risk of T2D

**Figure 6:**
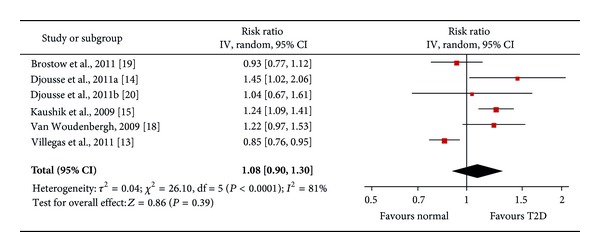
Forest plot of the meta-analysis for LC n-3 PUFA intake and incidence of type 2 diabetes.

The meta-analysis showed no significant association between high intake of marine LC n-3 PUFA (EPA and DHA) and incidence of T2D (pooled RR = 1.08, 95% CI: 0.90, 1.30, *P* = 0.39) (Figure 6). Significant heterogeneity (*I* ^2^ = 85%, *P* < 0.00001) caused by ethnicity and follow-up years in the trend level (*P* = 0.064, MWU test) could explain the inconsistency of the results. In the subgroup analysis, two studies [13, 19] in Asian populations with shorter follow-up time (<10 years) showed reduced risk of T2D with high intake of marine LC n-3 PUFA (pooled RR = 0.87, heterogeneity *P* = 0.42). Conversely, four studies [14, 15, 18, 20] with western participants and longer follow-up periods showed increased risk of T2D with increased LC n-3 PUFA consumption (pooled RR = 1.27, heterogeneity *P* = 0.7) (Table 2). Egger's regression test and funnel plot showed no significant publication bias (Egger's *P* > 0.05). Dose-response analysis for marine LC n-3 PUFA studies showed no significant dose-response relationship with the risk of T2D. 

## 4. Discussion

### 4.1. Heterogeneity Exploration and Risk of Bias

Meta-analysis allows us to increase the power of detecting associations between exposures and outcomes by increasing sample size. However, it may be complicated by study heterogeneity. Our subgroup analysis showed no significant source for the observed high heterogeneity between fish/seafood and marine LC n-3 PUFA studies, but ethnicity may be partially contributing to high heterogeneity. Two studies using Asian populations [13, 19] reported protective effects of LC n-3 PUFA intake on risk of T2D, while four studies using western populations [14, 15, 18, 20] showed opposite results. This may be related to differences in overall dietary patterns or genetic background between eastern and western populations.

Studies of different quality showed variable results. Two high quality studies [12, 13] showed a beneficial effect (RR = 0.87) of fish/seafood intake on risk of T2D with significance (*P* = 0.006). But it cannot be excluded that the beneficial effect of fish/seafood intake is related to ethnicity or fish/seafood consumption range instead of study quality because these two studies used Asian populations and had higher fish/seafood consumption (>80 g/day). Meanwhile, moderate quality fish/seafood studies yielded no significant conclusions and pointed to a pooled RR of 1.15. Publication bias (reporting bias) is unlikely because funnel plot and Egger's regression tests showed no significance, but other nonrandom biases are possible because of the failure to adjust some known T2D risk factors (e.g., physical activity, dietary pattern, age, BMI, or family history of diabetes) or lack of validation of the FFQ method. 

For the association between marine LC n-3 PUFA intake and risk of T2D, only one high quality study [13] showed a beneficial effect of marine LC n-3 PUFA intake on risk of T2D, while five studies with moderate quality showed a slightly increased risk pooled effect with weak significance (*P* = 0.04). Funnel plot and Egger's regression tests showed no significant publication bias for marine LC n-3 PUFA studies. However, there may be some nonrandom bias in moderate quality studies, as some confounding factors (such as family history of diabetes and dietary factors) were not well adjusted when calculating the RR. In the study of [19], for example, the RR may bias to risk because the vegetable/fruit intake (22.5 versus 14.3 g/100 kcal, highest versus lowest quartile) was not adjusted. 

Other possible sources of heterogeneity may include the amount of fish consumed, fish types, and gender. Two studies [12, 13] with more than 80 g/d of fish/seafood intake showed reduced incidence of T2D. Among six studies with less than 80 g/d fish/seafood intake, five showed either no effect or increased risk of T2D except for one study [16] that may be biased by a nonstandard outcome measurement. The range of EPA and DHA intake across studies may not explain the high heterogeneity in the marine LC n-3 PUFA studies (Table 1). Our dose-response analysis could reduce the bias caused by different doses of fish/seafood or marine LC n-3 PUFA intake set as the highest level within publications. The dose-response analysis for the fish/seafood intake studies showed a trend towards an inverse linear relationship between fish/seafood intake and risk of T2D, supporting that the range of fish/seafood intake may contribute to heterogeneity. The method of preparing the fish and the amount and type of fat added may also alter the effects of fish on glucose metabolism. There is only one included cohort study [16] that showed the effect of fried fish on risk of T2D (pooled RR = 0.91, 95% CI: from 0.75 to 1.10), which makes it in sufficient to conduct stratified meta-analysis according to the method of fish preparation. Most included prospective cohort studies used fish and seafood as exposure, which include fish (oily and lean fish) and other seafood (shellfish, octopus, and other fish products). This makes it difficult to clarify their relationships with T2D because different types of fish and seafood contain different ratios of nutrients and different levels of contaminants. More studies will be required to strengthen conclusions regarding the individual impact of fish versus seafood on T2D risk. 

### 4.2. Limitations

Publication bias is an important concern with meta-analysis. Although we found no significant publication bias in the current meta-analysis using Egger's test, the results should still be considered with caution. The statistical power for publication bias might be low because there are only ten studies for fish/seafood and six studies for marine n-3 PUFA in the meta-analysis. The likelihood of selection bias and recall bias is low because of the design of prospective studies, but the observational studies are limited because confounders were adjusted in different studies, making them difficult to compare. 

The method of dietary assessment may also affect the results. FFQ is related to random and systematic errors [26], which will underestimate the true associations between diet and diseases. Nutritional biomarkers in plasma should be monitored and will minimize this problem. One prospective study [19] also measured plasma levels of EPA and DHA, which were significantly associated with reduced risk of T2D (RR = 0.64 for EPA and DHA) in those with high plasma levels of EPA and DHA. However, three other studies showed no significant relationship between plasma/serum LC n-3 PUFA levels and risk of T2D [27–29].

### 4.3. Potential Mechanism Underlying the Beneficial Effects of Oily Fish Intake on Risk of T2D

Our current meta-analysis showed significant beneficial effect of oily fish intake on risk of T2D, corresponding to previous clinical trial findings that fish intake was associated with reduced fasting glycemia [30] and improved glucose tolerance [31]. One recent cross-sectional study in a Spanish population also showed that the high fish intake is related to low plasma level of glucose and low incidence of diabetes [32]. But ours and other 5 meta-analyses [33–37] showed no significant effects of fish intake on risk of T2D, although two prospective cohort studies [12, 13] showed that the beneficial effects of fish/seafood intake on risk of T2D, which used an Asian population, had high study quality and had high range of amount of fish intake (>80 g/d, highest versus lowest quartile). However high heterogeneity for fish/seafood studies remains to be clarified before any conclusions are to be made regarding the effects of high fish/seafood intake.

As oily fish has high amount of LC n-3 PUFA (EPA and DHA), we are wondering if EPA and DHA intake may contribute to the beneficial effect of oily fish intake. But ours and other 5 meta-analyses studies showed no significant association between LC n-3 PUFA intake and T2D risk. This is corresponding to one randomized double-blind placebo-controlled study which showed that fish oil has no significant effect on improving glucose control and insulin sensitivity in diabetic patients [38]. However, there are also contaminations in fish which may disrupt insulin signaling and glucose homeostasis, such as selenium and mercury [39, 40]. In the current meta-analysis, only one study [18] adjusted selenium level for RR and showed much lower RR after adjustment when measuring the effects of EPA and DHA intake. Nonetheless, there are other oily fish nutrients (such as vitamin D and fish protein) that may contribute to the beneficial effects of oily fish intake on T2D risk. Oily fish is a major diet source of vitamin D, and several recent cohort studies have demonstrated the protective effects of vitamin D on T2D incidence in various populations [41, 42]. 

## 5. Conclusions

Our meta-analysis showed no significant effect of fish/seafood or marine LC n-3 PUFA intake on risk of T2D. However high heterogeneity was found in the current meta-analysis, which may include the bias from different ethnicities, follow-up years, and amount of fish intake. In addition, our stratified meta-analysis showed a significant weak effect of oily fish intake on risk of T2D. Dose-response analysis suggested that 80 g per day intake of oily fish may reduce 20% risk of T2D. But no significant association between EPA and DHA intake and risk of T2D was found, suggesting that other nutrients from oily fish may contribute to the beneficial effects of oily fish intake, such as vitamin D and oily fish protein. However, more high quality prospective cohort studies will be needed to support our conclusion for beneficial effects of oily fish intake on T2D risk and to clarify the association between fish/seafood intake and marine LC n-3 PUFA intake and T2D incidence.

## Figures and Tables

**Table:** (a)

Fish/seafood	Ethnicity	Age	Follow-up years	Fish/seafood type	Fish/seafood consumption (highest versus lowest) (range)	Number of participants	Quartile	Adjustment for T2D risk factors	Quality score (0–12)	Adjusted RR (95% CI)
Nanri et al. 2011^A^	Japan	40–59	10	Total fish/seafood	166.6 g/d versus 35.9 g/d (130.7 g/d)	52680	4	Age, BMI, family history of diabetes mellitus, total physical activity, vegetable, fruit, and meat	11	0.84 (0.62–1.15)
Villegas et al. 2011^B^	China	40–74	4.1–8.9	Total fish/shellfish	99.2 g/d versus 14.0 g/d (85.2 g/d)	116129	5	Age, BMI, physical activity, family history of diabetes, and dietary pattern (vegetable, fruit, and meat)	11	0.87 (0.78–0.97)
Patel et al. 2009^C^	Norwich, England	40–79	7.5	Total fish/seafood	>14.28 g/d versus <14.28 g/d (14.28 g/d)	21984	2	Age, BMI, family history of diabetes, and physical activity	7	0.76 (0.59–0.96)
Patel et al. 2012^K^	Europe	55–63	6.9	Total fish/seafood	>51.8 g/d versus <15 g/d	24813	4	Age, BMI, physical activity, fruit, and vegetable intake	8	0.99 (0.86–1.15)
Montonen et al. 2005^J^	Finland	40–69	23	Total fish	>0 versus 0 g/d	4304	4	Age, BMI, and family history of diabetes	6	0.96 (0.71–1.29)
Schulze et al. 2003^D^	US	26–46	8	Total fish	>28.57 g/d versus <14.28 g/d (28.57 g/d)	91246	N/A	Age, BMI, physical activity, and family history of diabetes	8	1.04 (0.82–1.32)
Djoussé et al. 2011^E^	US	>45	12.4	Total fish/seafood	56.14 g/d versus 6.71 g/d (49.43 g/d)	36328	5	Age, BMI, parental history of diabetes, physical activity, and meat	8	1.49 (1.30–1.70)
Djoussé et al. 2011^F^	US	>65	15	Total fish	>71.43 g/d versus <3.3 g/d (71.43 g/d)	2831	5	Age, BMI, and physical activity	6	1.07 (0.35, 3.33)
Kaushik et al. 2009^G^	US	26–78	14.5–18.5	Total fish (finfish)	>71.43 g/d versus <3.3 g/d (78.57 g/d)	195204	5	Physical activity, family history of diabetes mellitus, and BMI	7	1.22 (1.08–1.38)
Van Woudenbergh et al. 2009^H^	Dutch	>55	12	Total fish/seafood	35.6 g/d versus 0 g/d (35.6 g/d)	4472	4	Age	5	1.32 (1.02–1.70)

**Table:** (b)

Fish	Ethnicity	Age	Follow-up years	Fish type	Fish consumption	Number of participants	Quartile	Adjustment for T2D risk factors	Quality score	Adjusted RR (95% CI)
Nanri et al. 2011^A^	Japan	40–59	10	Fresh fish	95.4 g/d versus 13.6 g/d (81.8 g/d)	52680	4	Age, BMI, family history of diabetes mellitus, physical activity, vegetable, fruit, and meat	11	0.91 (0.73–1.13)
Villegas et al. 2011^B^	China	40–74	4.1–8.9	Fish	79.6 g/d versus 9.6 g/d (70 g/d)	116156	5	Age, BMI, physical activity, family history of diabetes, and dietary pattern (vegetable, fruit, and meat)	11	0.90 (0.80–1.01)
Kaushik et al. 2009^G^	US	26–78	14.5–18.5	Finfish	>71.43 g/d versus <3.3 g/d (78.57 g/d)	195204	5	Physical activity, family history of diabetes, and BMI	7	1.22 (1.08–1.38)
Montonen et al. 2005^J^	Finland	40–69	23	Total fish	>0 versus 0 g/d	4304	4	Age, BMI, and family history of diabetes	6	0.96 (0.71–1.29)
Patel et al. 2009^C^	Norwich, England	40–79	7.5	White fish, oily fish	>14.28 g/d versus <14.28 g/d (14.28 g/d)	21984	2	Age, BMI, family history of diabetes, and physical activity	7	0.90 (0.79–1.03)
Patel et al. 2012^K^	Europe	55–63	6.9	Total fish	>34.9 g/d versus <2.9 g/d	24813	4	Age, BMI, physical activity, fruit, and vegetable intake	8	1.06 (0.92–1.22)
Van Woudenbergh et al. 2009^H^	Dutch	>55	12	Lean fish, fatty fish	46.3 g/d versus 0 (46.3 g/d)	4472	4	Age	5	1.16 (0.89–1.51)

**Table:** (c)

LC n-3 PUFA	Ethnicity	Age	Follow-up years	LC n-3 PUFA type	LC n-3 PUFA consumption	Number of participants	Quartile	Adjustment for T2D risk factors	Quality score	Adjusted RR (95% CI)
Villegas et al. 2011^B^	China	40–74	4.1–8.9	EPA, DHA	0.2 g/d versus 0.02 g/d (0.18 g/d)	64193	5	Age, BMI, physical activity, family history of diabetes, and dietary pattern (vegetable, fruit, and meat)	11	0.85 (0.76–0.95)
Brostow et al. 2011^I^	Singapore, Chinese	45–74	6	EPA, DHA	0.6 g/d versus 0.11 g/d (0.49 g/d)	43175	5	Age, BMI, and physical activity	7	0.93 (0.77–1.11)
Kaushik et al. 2009^G^	US	26–78	14.5–18.5	EPA, DHA	0.46 g/d versus 0.07 g/d (0.37 g/d)	195204	5	Physical activity, family history of diabetes, and BMI	7	1.24 (1.09–1.41)
Djoussé et al. 2011^E^	US	>45	12.4	EPA, DHA	0.39 g/d versus 0.05 g/d (0.34 g/d)	36328	5	Age, BMI, parental history of diabetes, physical activity, and meat	8	1.45 (1.32–1.59)
Djoussé et al. 2011^F^	US	>65	15	EPA, DHA	>0.56 g/d versus <0.17 g/d (0.6 g/d)	3088	4	Age, BMI, and physical activity	6	1.04 (0.67–1.61)
Van Woudenbergh et al. 2009^H^	Dutch	>55	12	EPA, DHA	0.237 g/d versus 0.024 g/d (0.21 g/d)	4472	3	Age	5	1.22 (0.97–1.53)

A–I: A: T2D was assessed by Japanese criteria [12]; B: T2D was assessed by ADA criteria [13]; C: T2D was assessed by other resource and hospital records [16]; D: T2D was assessed by NDDG criteria [17]; E: T2D was assessed by ADA criteria [14]; F: T2D was assessed by medication use and fasting/nonfasting glucose level in plasma [20]; G: T2D was assessed by NDDG criteria [15]; H: T2D was assessed by ADA/WHO criteria [18]; I: T2D was self-reported [19]; J: T2D was assessed by physician according to diabetic criteria [22]; K: T2D was assessed by multiple sources [21].

**Table 2:** Subgroup analysis to investigate heterogeneity source in meta-analysis.

Fish/seafood intake	Publications	Pooled RR, ^1^ *P* value	Heterogeneity ^2^ *P* value	^ 3^ *P* value	Marine LC n-3 PUFA	Publications	Pooled RR, ^1^ *P* value	Heterogeneity ^2^ *P* value	^ 3^ *P* value
*Ethnicity *				0.18	*Ethnicity *				0.064
Asian (Chinese, Japanese)	2 [12, 13]	0.87, 0.006	0.83		Asian (Chinese, Japanese)	2 [13, 19]	0.87, 0.005	0.42	
US/European	8 [14–18, 20–22]	1.10, 0.22	0.0001		US/European	4 [14, 15, 18, 20]	1.27, <0.00001	0.7	
*Length of follow-up (years) *				0.17	*Length of follow-up (years) *				0.064
<10	4 [13, 16, 17, 21]	0.91, 0.11	0.16		<10	2 [13, 19]	0.87, 0.005	0.42	
≥10	6 [13–15, 18, 20, 22]	1.17, 0.04	0.02		≥10	4 [14, 15, 18, 20]	1.27, <0.00001	0.7	
*Assessment of T2D *				0.18	*Assessment of T2D *				NA
Confirmed by physician/phone interview/hospital records	3 [16, 21, 22]	0.91, 0.28	0.2		Confirmed by physician/phone interview/hospital records	0	NA	NA	
Confirmed by standard criteria or plasma glucose measurement	7 [12–15, 17, 18, 20]	1.10, 0.31	<0.0001		Confirmed by standard criteria or plasma glucose measurement	6 [13–15, 18–20]	1.09, 0.36	<0.00001	
*Sample size *				0.51	*Sample size *				1
<10000	3 [18, 20, 22]	1.14, 0.26	0.29		<10000	2 [18, 20]	1.18, 0.11	0.53	
≥10000	7 [12–17, 21]	1.01, 0.88	<0.0001		≥10000	4 [13–15, 19]	1.07, 0.57	<0.0001	
*Quality score *				0.18	*Quality score *				0.33
High (9–12)	2 [12, 13]	0.87, 0.006	0.83		High (9–12)	1 [13]	0.85, NA	NA	
Moderate (5–8)	8 [14–18, 20–22]	1.1, 0.22	0.0001		Moderate (5–8)	5 [14, 15, 18–20][16, 17, 20–22]	1.17, 0.01	0.03	

^1^ *P* value calculated by *Z* statistic.

^2^ *P* value calculated by *I* ^2^ statistic.

^3^ *P* value calculated by Mann-Whitney *U* test.
